# Efficacy and Safety of Xiaoyao Recipe in the Treatment of Poststroke Depression: A Systematic Review and Meta-Analysis

**DOI:** 10.1155/2022/4385783

**Published:** 2022-04-13

**Authors:** Dou Wang, Tao Li, Jie Ron, Meiling Mao, Yifan Yang, Yalun Feng, Yongmei Yan

**Affiliations:** ^1^Affiliated Hospital of Shaanxi University of Traditional Chinese Medicine, Xianyang 712000, China; ^2^First Clinical Medical College, Shaanxi University of Traditional Chinese Medicine, Xianyang 712000, China

## Abstract

**Background:**

Poststroke depression (PSD) is a common neuropsychiatric disorder that affects the disability, mortality, functional recovery, and quality of daily life of patients. Xiaoyao Recipe (XYR) is often used to treat PSD and has achieved good clinical effects, but it lacks reliable evidence.

**Objective:**

This study aims to evaluate the effectiveness and safety of XYR on PSD through meta-analysis.

**Methods:**

A comprehensive literature search was carried out in multiple databases, including PubMed, the Cochrane Library, Chinese Biomedical Literature Service System, China National Knowledge Infrastructure, Wanfang Database, VIP Database, and ClinicalTrials, from inception to July 1, 2021, to collect randomized controlled trials that applied XYR for patients with PSD. For a controlled trial, the search time limit was set from the time of the database's establishment to July 2021. Two experienced researchers independently screened the literature according to the inclusion and exclusion criteria, extracted data, evaluated the quality of the literature, and used RevMan 5.3 software for meta-analysis.

**Results:**

A total of 12 studies were included in this study, involving 882 patients with PSD who were hospitalized or outpatients. The meta-analysis results showed that the total effective rate (*p* < 0.00001) of the test group (XYR or XYR combined with antidepressants) after treatment was high; Hamilton's Depression Scale score (*p* < 0.000001), Scandinavian Stroke Scale score (*p*=0.004 < 0.05), and Barthel index (*p* < 0.00001) were improved; the incidence of adverse reactions (*p* < 0.00001) was low; and the serum serotonin content (*p* < 0.00001) was high.

**Conclusion:**

Compared with antidepressant drugs, XYR is more effective and safer in the treatment of PSD patients. However, more high-quality studies are needed to further support the above conclusions.

## 1. Introduction

Poststroke depression (PSD) refers to a severe neuropsychiatric disorder that occurs after a stroke, showing a series of affective disorder syndromes characterized by low mood, decreased interest, apathy, and pessimism in addition to stroke symptoms. Moreover, it is often accompanied by physical symptoms such as fatigue, pain, palpitation, and loss of appetite [1]. The incidence of PSD can reach 30%–50% [2, 3]. PSD not only hinders the recovery of neurological function but also severely affects the activities of daily living and even increases the disability and mortality of patients [4–6]. The pathogenesis of PSD involves endogenous mechanisms such as lack of monoamine transmitters, insufficient levels of neurotrophic factors, hypothalamic-pituitary-adrenal (HPA) axis dysfunction, neuroinflammation, and other endogenous mechanisms [7, 8]. It is also related to poststroke psychological, family, and social reactions. The current treatment methods for PSD mainly include drugs and nondrug therapies (such as acupuncture, rehabilitation, psychology, music, exercise, and physical therapy).

The clinical medications for PSD include selective serotonin reuptake inhibitors and tricyclic antidepressants. Studies have shown that these antidepressants can effectively improve depression symptoms [9–12]. Long-term use of depression drugs can easily cause side effects such as gastrointestinal dysfunction, neurological symptoms, and mental disorders, and may even increase the risk of cardiovascular and cerebrovascular event [7, 13]. In recent years, traditional Chinese medicine has often been used to treat PSD. Xiaoyao Recipe (XYR) is a classic Chinese herbal medicine used in the treatment of liver depression and spleen deficiency syndrome. XYR is a compound composed of eight kinds of herbs: *Bupleurum*, Radix Paeoniae Rubra, *Angelica*, Poria, *Atractylodes macrocephala*, ginger, peppermint, and licorice. It has the effects of soothing the liver and relieving depression, nourishing the blood and softening the liver, invigorating the spleen, and replenishing qi. It is widely used in the treatment of depression, PSD, chronic hepatitis, chronic gastritis, gastrointestinal neurosis, duodenal ulcer, breast lobular hyperplasia, and cholelithiasis.

Several randomized controlled studies have shown that XYR alone or in combination with antidepressants can improve PSD symptoms [14, 15]. Because few large-sample randomized controlled trials (RCTs) using XYR have been conducted on the PSD population, a reliable evidence-based basis regarding the use of XYR in treating PSD is still lacking. Several previous meta-analyses have suggested a beneficial effect on depression, of which few of meta-analyses explored the effect of XYR on PSD [16, 17]. Only one meta-analysis explored the effect of XYR on PSD, but this study included fewer randomized controlled studies, failed to conduct subgroup analysis of different interventions, and the outcome indicators were not comprehensive [15]. Therefore, we used published randomized controlled studies on the effect of XYR on depressive symptoms in PSD patients for our meta-analysis. The outcome measures included total effective rate, Hamilton's Depression Scale (HAMD), and adverse reaction rate. This study aims to comprehensively evaluate the efficacy and safety of XYR in the treatment of PSD through meta-analysis, and the results will provide more effective and reliable evidence for clinicians, researchers, and healthcare policy makers. Therefore, this study intends to systematically evaluate the clinical efficacy and safety of XYR in the treatment of PSD and conduct a meta-analysis of existing RCTs.

## 2. Methods

This systematic review strictly follows the guidance of the Preferred Reporting Items for Systematic Review and Meta-Analysis Protocols. The research protocol has been published on PROSPERO (CRD42020203349).

### 2.1. Search Strategy

Databases including PubMed, the Cochrane Library, Chinese Biomedical Literature Service System, China National Knowledge Infrastructure, Wanfang Database, and VIP Database were searched. The search time range was from the establishment of the database to July 2021. A combination of free words and subject words was used in the search. The search terms included “Xiaoyao San,” “Xiaoyao Pill,” “Xiaoyao Recipe,” “Stroke,” “Depression,” and “Depression after Stroke.”

### 2.2. Inclusion and Exclusion Criteria

#### 2.2.1. Inclusion Criteria

We used the following research inclusion criteria:

1. Study type: this study will include all RCTs for XYR treatment of PSD patients
2. Participant type: all patients diagnosed with PSD, regardless of nationality, age, gender, stroke type, and location
3. Type of intervention: the experimental group used XYR or XYR combined with antidepressants as interventions, and the control group used placebo or antidepressants alone
4. Outcome indicators: the main outcome indicators are as follows: (1) total effective rate; (2) Hamilton's Depression Scale (HAMD). Secondary outcome indicators are as follows: (1) adverse reaction rate; (2) Scandinavian Stroke Scale (SSS); (3) Barthel index (BI) for activities of daily living; and (4) serum serotonin (5-HT) content.

#### 2.2.2. Exclusion Criteria

1. Conference papers, abstracts, animal experiments, and reviews
2. Repeated publications of the same research population
3. Journal articles published by a single author
4. Unable to obtain complete data in the literature

### 2.3. Data Extraction

Two researchers independently screened the literature, extracted the data, and performed cross-checking. If they encountered a disagreement, they resolved it through discussion or negotiation with a third reviewer. When selecting documents, the title and abstract were first read. After excluding obviously irrelevant documents, the full text was read to determine whether to include them. The content of data extraction includes: (1) basic information of the included research: research title, author, publication year, and journal name; (2) baseline characteristics of the research object; (3) intervention measures; and (4) outcome indicators.

### 2.4. Quality Assessment

Two researchers independently evaluated the risk of bias in the included literature and cross-checked the results. The risk of bias tool described in the Cochrane systematic review manual was used to evaluate the literature included in the RCTs. The evaluation content included the following six aspects: random sequence generation, allocation hiding, blinding of result evaluation, blinding of participants and researchers, incompleteness of result data, and selective reporting of research results and other biases. The evaluation results are divided into three levels: “unclear judgment risk,” “low bias risk,” and “high bias risk.”

### 2.5. Data Analysis

A meta-analysis was performed using RevMan 5.3 software. The included studies were tested for heterogeneity, and the *x*^2^ test was used for analysis (the test level was *α* = 0.1). The degree of heterogeneity was quantitatively judged by combining with *I*^2^. *I*^2^ < 50% and *p* > 0.1 indicate a small heterogeneity, and the fixed effects model is adopted. *I*^*2*^ ≥ 50% and *p* ≤ 0.1 indicate a large heterogeneity, and the random effects model is used for analysis. When the measurement tool and the unit of the continuous variable are the same, the standardized mean difference (SMD) is used. When the measurement tools or units of continuous variables are not the same, the weighted average difference is used. The risk ratio (RR) is used as the effect analysis statistic for binary variables. All effect sizes are provided with 95% confidence intervals (95% CI). If the required research data are not reported in the study, the researcher will contact the original author via phone or e-mail to obtain additional information. If the required research data are not available, we will use descriptive analysis or exclude these studies when necessary.

### 2.6. Sensitivity Analysis

We used the one-by-one elimination method to analyze the sensitivity of the research results.

### 2.7. Deviations in Assessment Reports

If the number of included studies is greater than 10, we will draw a funnel chart to assess whether there is publication bias. If the funnel chart is symmetrical, no publication bias is present.

## 3. Results

### 3.1. Search Results

**Figure 1:**
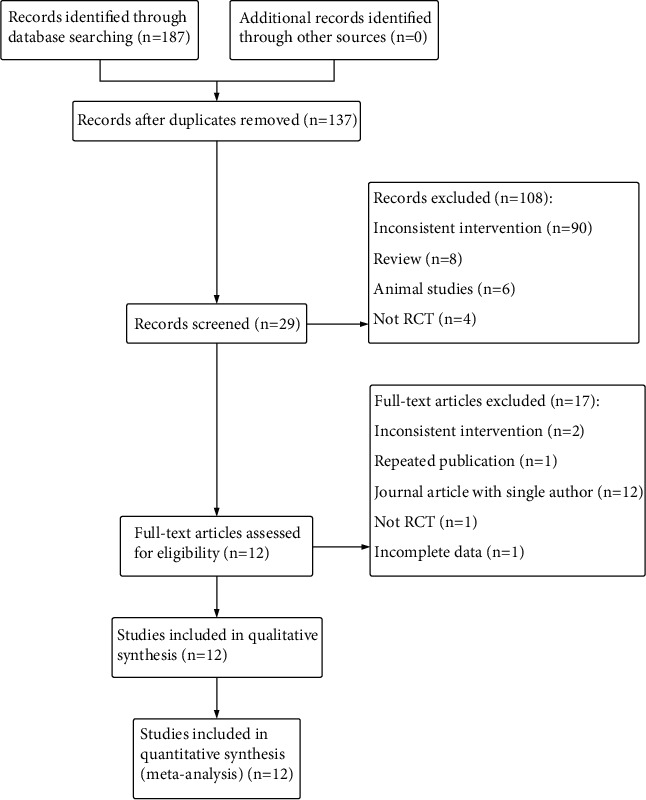
Flow diagram of literature screening.

A total of 187 references were obtained from the preliminary search, and 137 were obtained after removing duplicates (Figure 1). Two authors independently screened these references. After reading the titles and abstracts, 108 articles were excluded, and the remaining 29 full texts were reviewed. Thereafter, 17 articles were excluded, and 12 eligible articles were included. These 12 studies were conducted in China and published between 2004 and 2020. The basic characteristics of the included studies are shown in Tables 1 and 2, involving a total of 882 PSD patients from hospitalization or outpatient clinics.

### 3.2. Risk of Bias Assessment

**Figure 2:**
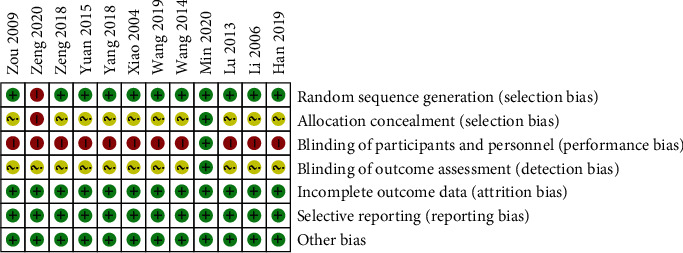
Summary of risk of bias.

Two investigators independently used the RCT risk of bias tool recommended in Cochrane Handbook 5.1.0 to evaluate the quality of the included studies. One study [18] did not mention randomization, and 11 studies correctly used randomization methods, including 4 studies [18–21] that described specific randomization methods. One study [18] reported allocation concealment and implemented blinding, and the remaining studies did not report allocation concealment and did not use blinding. All studies did not report the loss of follow-up and withdrawal of the study subjects, but the study data report was complete. Although all studies did not report whether the plan was set in advance, they reported in detail that the baseline situation was comparable. The detailed results are shown in Figure 2.

### 3.3. Meta-Analysis Results

Of the 12 included studies, 3 studies [18, 22, 23] compared oral XYR alone with antidepressants, and 9 studies [19, 20, 22, 24–29] compared the combination of oral XYR and antidepressants with antidepressants.

#### 3.3.1. Total Efficiency Rate

**Figure 3:**
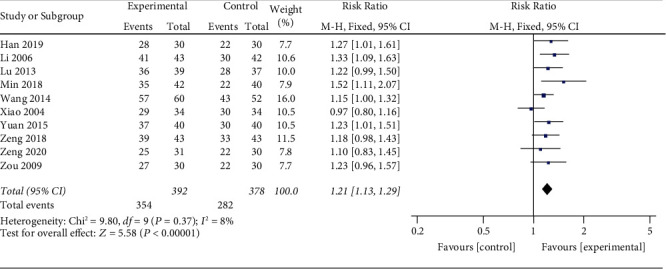
Meta-analysis of XYR and the total effective rate of antidepressants.

10 studies [18–23, 26–29] reported the total effective rate (Figure 3). The heterogeneity test indicated that the studies were not heterogeneous (*p*=0.37; *I*^2^ = 8%). Meta-analysis of the research data using a fixed effects model showed that the total effective rate of the experimental group was better than that of the control group, and the results showed significant statistical differences (RR = 1.21; 95% CI: 1.13, 1.29; *p* < 0.00001).

#### 3.3.2. HAMD Score

**Figure 4:**
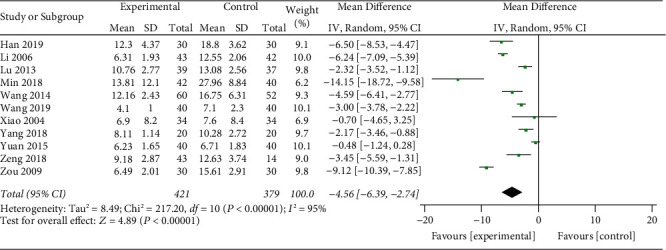
Meta-analysis of XYR and HAMD scores for antidepressants.

11 studies [18–22, 24–29] reported the HAMD score (Figure 4). The heterogeneity test indicated significant heterogeneity in each study (*p* < 0.00001; *I*^2^ = 95%). However, the forest plot and the confidence interval are to the left of the invalid line, indicating that the heterogeneity between studies does not affect the results. Therefore, the random effects model was selected for the meta-analysis, which showed that the HAMD score of the test group was better than that of the control group. The results were statistically significant (MD: − 4.56; 95% CI: −6.39,−2.74; *p* < 0.000001). The reasons for heterogeneity may be related to differences in intervention measures, antidepressants, and treatment courses between studies.

#### 3.3.3. Adverse Reaction Rate

**Figure 5:**
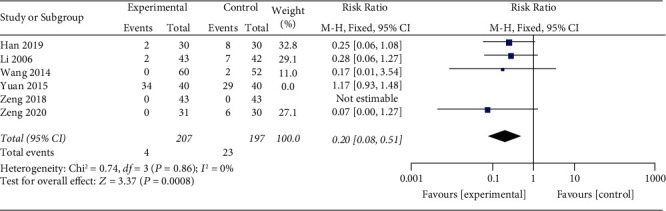
Meta-analysis of adverse reaction rates between XYR and antidepressants.

The heterogeneity test showed significant heterogeneity (*p*=0.0001; *I*^2^ = 83%). A one-by-one elimination method was used to analyze the source of heterogeneity. When the Yuan study [21] was excluded, the heterogeneity was significantly reduced (*p*=0.86; *I*^2^ = 0%), indicating that the study is a source of heterogeneity (Figure 5). After excluding heterogeneity, a fixed effects model was used to conduct a meta-analysis on the research data [19, 20, 23–26, 29]. The results showed that the incidence of adverse reactions in the experimental group was lower than that in the control group, and the results were statistically different (RR = 0.20; 95% CI: 0.08, 0.51; *p* < 0.00001).

#### 3.3.4. SSS Score

**Figure 6:**
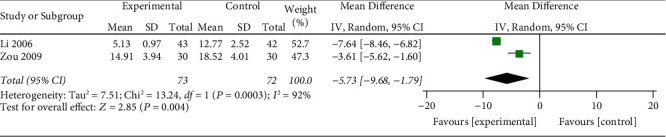
Meta-analysis of XYR and antidepressant SSS scores.

2 studies [28, 29] reported SSS scores (Figure 6). The heterogeneity test showed significant heterogeneity (*p*=0.0003; *I*^2^ = 92%). The reasons for this heterogeneity could not be further analyzed due to the small number of included studies. The research results are all on the left side of the invalid line. The meta-analysis of the research data using the random effects model showed that the experimental group is better than the control group with a significant statistical difference (MD: − 5.73; 95% CI: −9.86, −1.79; *p*=0.004).

#### 3.3.5. BI

**Figure 7:**
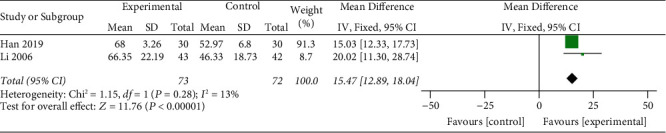
Meta-analysis of XYR and BI index of antidepressants.

2 studies [20, 29] reported on the BI (Figure 7). The heterogeneity test indicated that the studies were not heterogeneous (*p*=0.28; *I*^2^ = 13%). The meta-analysis of the research data using a fixed effect model showed that the BI score of the experimental group was better than that of the control group, and the results had significant statistical differences (MD = 15.47; 95% CI: 12.89, 18.04; *p* < 0.00001).

#### 3.3.6. Serum 5-HT Content

**Figure 8:**
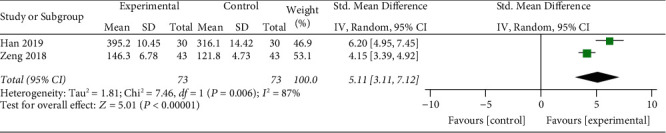
Meta-analysis of XYR and antidepressant serum 5-HT levels.

2 studies [19, 20] reported serum 5-HT levels (Figure 8). The heterogeneity test indicated significant heterogeneity in each study (*p*=0.006; *I*^2^ = 87%). The reasons for this heterogeneity could not be further analyzed due to the small number of included studies. The research results are all on the side of the invalid line. A meta-analysis of the research data using a random effects model showed that the serum 5-HT content of the test group was higher than that of the control group, and the difference was statistically significant (SMD: 5.11; 95% CI: 3.11, 7.12; *p* < 0.00001).

### 3.4. Sensitivity Analysis

The abovementioned outcome indicators were all eliminated one by one for sensitivity analysis. After eliminating the included studies one by one, the change in effect size and *p*value was small. This shows that the results of the meta-analysis are stable and credible.

### 3.5. Publication Bias

**Figure 9:**
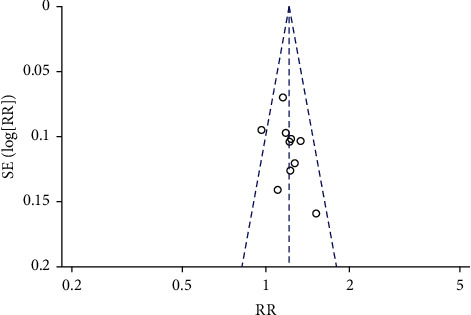
The total effective rate of publication bias funnel chart.

**Figure 10:**
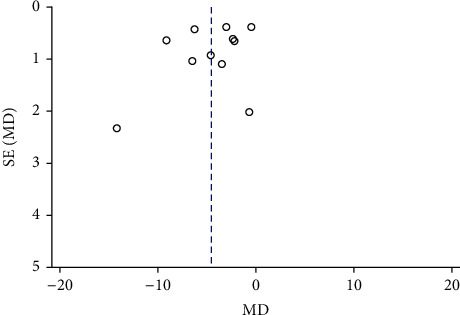
Funnel chart of publication bias of HAMD score.

The funnel chart was created with the total effective rate [18–23, 26–29] (Figure 9) and the HAMD score [18–22, 24–29] (Figure 10). The graph is not completely symmetrical, indicating that the included studies may have publication bias. This may be due to the fact that some studies with negative results have not been published, the sample size of the included studies is small, and the total number of included studies is small.

## 4. Discussion

This study is a meta-analysis of the effectiveness and safety of XYR in the treatment of PSD. After treatment, the total effective rate and HAMD score of the test group were better than those of the control group, indicating that XYR alone or in combination with antidepressants is better than antidepressant therapy alone in relieving PSD. The incidence of adverse reactions in the experimental group was significantly lower than that in the control group, proving the safety of XYR. The SSS score can reflect the prognosis of stroke, and the BI can measure the patient's activities of daily living [30]. The two are obviously related. The higher the SSS score, the lower the BI. The SSS score and BI of the experimental group were better than those of the control group, but this result is derived from a few studies. The above results suggest that XYR treatment of PSD not only has better clinical efficacy, but also has higher safety than antidepressant treatment.

The clinical incidence of PSD is seriously underestimated. The following reasons may jointly lead to a high rate of missed diagnosis of PSD. First of all, PSD is easily misunderstood by patients and their families as a psychological burden caused by stroke. Second, the symptoms of aphasia and cognitive impairment caused by stroke may conceal the symptoms of PSD. Finally, and most importantly, the clinical diagnosis of PSD lacks specific laboratory indicators. In addition, PSD is significantly related to the prognosis of stroke. Compared with a simple stroke, patients with PSD have obstacles to recovery of neurological function, a significant decrease in their quality of life, and a significant increase in mortality [31]. Interventions in the process of PSD can not only improve the symptoms of depression but also help the recovery of stroke, and their benefits are far greater than the treatment of depression alone. The occurrence of PSD is not only related to stroke brain damage and accompanying cognitive impairment, functional disability, and reduced quality of life, but also related to social and psychological factors such as past history of affective disorders, personality characteristics, coping styles, and social support [32, 33]. Therefore, in the treatment of PSD, a variety of treatment methods such as drug therapy, psychotherapy, and rehabilitation training should be used in order to achieve the best therapeutic effect. The goal of medical treatment of PSD is to relieve symptoms, improve quality of life, and prevent recurrence. During the treatment process, attention should be paid to monitoring and evaluating the efficacy, compliance, adverse reactions, and the possibility of recurrence of symptoms. As a Chinese herbal compound with good clinical efficacy and high safety, XYR may meet the clinical diagnosis and treatment needs of PSD.

The pathogenesis of PSD involves multiple levels of physiology, psychology, and society. The clinical symptoms are diverse. Therefore, drugs with a single target and mechanism may have limitations in their efficacy. Traditional Chinese medicine conducts syndrome differentiation and treatment from a holistic view. In the case of accurate syndrome differentiation, Chinese herbal medicine has the advantages of remarkable curative effect, strong individualization, low toxic and side effects, good patient compliance, and multilevel efficacy. XYR is a classic prescription in Chinese medicine for treating emotional diseases. Clinical studies and animal experiments have found that XYR has significant antidepressant effects [14]. XYR can improve the content of monoamine neurotransmitters in the hippocampus and cortex, restore the negative feedback function of the HPA axis, inhibit the expression of inflammatory factors, and adjust the abnormal level of brain–gut peptides by interfering with key molecules of the BDNF/CREB signal pathway in the hippocampus and cortex [34–37]. It improves depression through the nerve-endocrine-immune axis pathway. Bupleurum and Radix Paeoniae Alba are the key medicines prescribed for many treatments of emotional or mental illness. Traditional Chinese medicine believes that Bupleurum can soothe the liver and relieve depression, and white peony can nourish the yin and soften the liver. Modern pharmacological studies have shown that Bupleurum extract can inhibit inflammatory factors, reduce neuronal apoptosis, increase brain-derived nerve growth factor, and regulate HPA axis function to have an antidepressant effect [38–40]. In addition to inhibiting inflammatory factors by acting on monoamine neurotransmitters, paeoniflorin has an antidepressant effect, and it also has a certain protective effect on neuronal damage [41, 42]. Of note, the combination of *Bupleurum* and peony can exert an enhanced antidepressant effect [43].

This study has several limitations. First of all, most of the studies included did not provide sufficient detailed methodological information, such as allocation concealment methods and blinding information. Second, the studies included may have publication bias. This is related to the fact that positive results are easier to publish, and there may be an overestimation of the impact. Finally, the included studies are heterogeneous in the meta-analysis of some indicators. We used a random effects model to ensure that the studies had uniform weights. The heterogeneity of the study may be related to the disease course, intervention drugs, and treatment courses of the patients included in the study.

## 5. Conclusion

According to the evidence provided in this study, XYR can also be used to treat PSD on the basis of simple depression in the past, especially for patients with poor response to antidepressants or severe side effects. However, a strictly designed RCT is needed to support the clinical therapeutic effect of XYR treatment on PSD.

## Figures and Tables

**Table 1:** Basic characteristics of the included studies.

Study	No. (T/C)	Gender		Age		Outcome
		T	C	T	C	
Han 2019	30/30	18/12	16/14	56.13 ± 8.21	55.77 ± 7.16	①②③⑤⑥
Li 2006	43/42	23/20	22/20	69.53 ± 7.87	68.23 ± 7.35	①②③⑤④
Lu 2013	39/37	24/15	22/15	56.28 ± 2.14	57.23 ± 1.92	①②
Min 2018	42/40	22/20	22/18	64.24 ± 9.86	63.28 + 10.2	①②
Wang 2014	60/52	35/25	33/19	55.1	56.7	①②③
Wang 2019	40/40	20/20	27/13	55 ± 1.4	55.5 ± 1.5	②
Xiao 2004	34/34	—	—	—	—	①②
Yang 2018	20/20	10/10	11/9	53.83 ± 3.9	54.32 ± 4.3	②
Yuan 2015	40/40	24/16	22/18	49.05 ± 6.25	49.85 ± 5.85	①②③
Zeng 2018	43/43	23/20	25/18	57.53 ± 4.7	59.1 ± 5.33	①②③⑥
Zeng 2020	31/30	15/16	14/16	64.35 ± 7.16	64.8 ± 7.08	①③
Zou 2009	30/30	18/12	19/11	67.9 ± 6.1	66.8 ± 7.1	①②④

*Note.* ①effective rate; ②HAMD; ③adverse reaction rate; ④SSS; ⑤BI; ⑥serum 5-HT content.

**Table 2:** Characteristics of interventions.

Study	Course (days)	Interventions	
		T	C
Han 2019	28	Xiaoyao decoction, bid; flupentixol and melitracen tablets, 1^#^ bid	Flupentixol and melitracen tablets, 1^#^ bid
Li 2006	56	Xiaoyao decoction, bid; fluoxetine, 20 mg qd	Fluoxetine, 20 mg qd
Lu 2013	28	Xiaoyao decoction, bid; paroxetine, 20 mg qd	Paroxetine, 20 mg qd
Min 2018	90	Xiaoyao pill, 6 g bid	Fluoxetine, 10 mg bid
Wang 2014	14	Xiaoyao pill, 8^#^ tid; flupentixol and melitracen tablets, 1^#^ bid	Flupentixol and melitracen tablets, 1^#^ bid
Wang 2019	60	Xiaoyao decoction, bid; escitalopram, 20 mg bid	Escitalopram, 20 mg bid
Xiao 2004	42	Xiaoyao decoction, bid	Fluoxetine, 20–40 mg qd
Yang 2018	56	Xiaoyao decoction, bid; sertraline, 25 mg qd	Sertraline, 25 mg qd
Yuan 2015	28	Xiaoyao decoction, bid; escitalopram, 10 mg qd	Escitalopram, 10 mg qd
Zeng 2018	28	Xiaoyao pill, 8^#^ tid; fluoxetine, 20 mg qd	Fluoxetine, 20 mg qd
Zeng 2020	42	Xiaoyao decoction, bid	Fluoxetine, 20 mg qd
Zou 2009	42	Xiaoyao pill, 8# tid; flupentixol and melitracen tablets, 1^#^ bid	Flupentixol and melitracen tablets, 1^#^ bid

## Data Availability

The data set used in the current study can be obtained from the corresponding author based on reasonable requirements.

## Abbreviations

PSD: Poststroke depression
XYR: Xiaoyao recipe
RCT: Randomized controlled trial
HAMD: Hamilton Depression Scale
SSS: Edinburgh-Scandinavian Stroke Scale
BI: Barthel Index
SMD: Standardized mean difference
WMD: Weighted average difference
RR: Risk ratio
95% CI: 95% confidence interval.
